# ‘Solitude is not thrust upon any lovable person’: loneliness, shame, and the problem (of) personality

**DOI:** 10.1332/14786737Y2023D000000004

**Published:** 2024-04

**Authors:** Fred Cooper

**Affiliations:** Wellcome Centre for Cultures and Environments of Health, University of Exeter

In the early 1950s, a reformist philanthropic society, the Women’s Group on Public Welfare (WGPW), invited a series of experts to address their members on the theme of loneliness. This marked a period of research and consultation which would later result in a 1957 report published by the National Council of Social Service (National Council of Social Service 1957). While the report itself is a rich historical artefact of understandings of loneliness in post-war Britain, the archive of the WGPW offers considerable context and depth; not least in recording how a diverse group of psychiatrists, psychologists, academics, doctors, social workers, and housing officers spoke to the topic under consideration (Papers of the Women’s Forum and its Predecessors: London School of Economics Archive). Repeatedly, these expert testimonies addressed the problem of personality. The WGPW’s first speaker, the medical director of the Roffey Park Rehabilitation Centre, T.M. Ling, identified loneliness as the ‘chief problem of the mid-twentieth century.’ Although he implicated a number of (largely nostalgic) social and cultural factors, such as a supposed decline in neighbourliness and religious observance, Ling argued that efforts to mitigate loneliness through initiatives such as community centres and social clubs were frequently frustrated by the very people they targeted: ‘often the lonely person was the difficult person, their very way of life contributing to the fact that they did not fit in easily with other people’ (Ling 1954). A second speaker, the self-help author Peggy Makins (who wrote and spoke under the pen name Evelyn Home), framed the point in less tactful terms: ‘as we are trying to get to the roots of this problem, it should be made clear from the outset that the lonely person is fundamentally very unlovable… loneliness is solitude thrust upon us and solitude is not thrust upon any lovable person’ (Home 1955).

The relationship between loneliness and shame is frequently asserted, usually under-theorised, and very rarely subject to historical attention and care. Researchers in and across a number of disciplines are comfortable in the truism that shame and stigma are attached to loneliness through a series of psychosocial processes and dialogues, with negative social, medical, cultural, and political valuations of loneliness dovetailing into neoliberal (and older) logics of individual responsibility for relationships and health (Dolezal 2022). People who experience loneliness are frequently made to feel ashamed, as structural causative stories (such as the cost of living crisis, the impact of austerity policies, or the longer and deeper unravelling of the welfare state) are elided in favour of a shaming language of social ineptness and personal failings (Stenning and Hall 2018; Magnet and Orr 2022). As the cultural and social geographer Eleanor Wilkinson recently put it:

Contemporary constructions often position loneliness as something shameful and potentially pathological. Loneliness is framed as an individual failure, or a failure of community, rather than a structural condition. While loneliness is now understood as widespread, there is still a sense that one should not admit to feeling lonely, as to be lonely is to have failed

(Wilkinson 2022).

Indeed, participating in a recent UK study, one young person suggested that ‘to be seen as lonely is to be seen as though there’s something wrong with you.’ This study also found that 81% of the young people surveyed cited fear of other people’s reactions as a barrier to speaking about loneliness (Co-op 2019, 8). This evidence mirrored earlier research with young people by the same organisation, in which participants expressed the fear that if they were to come forward to seek help for their feelings of loneliness, they would simply be told to ‘pull themselves together’ (Co-op 2016, 27). Without entertaining the analytically useless assumption that loneliness is an *intrinsically* shaming experience, it can be difficult – particularly in the contexts noted above – for us not to interpret the presence, absence, or health of our relationships as signifiers of individual worth. In his 2021 book *Allein* (Alone), for example, the German essayist Daniel Schreiber described ‘der Glaube, dass ich nicht wirklich geliebt werden könne, nicht im eigentlichen Sinne liebenswert sei’: ‘the belief that I could not really be loved, was not, in any real sense, lovable’ (Schreiber 2021).

Read alongside one another, Schreiber and Home’s words draw focus to a historical – and ongoing – tension in how we address questions over ‘lovability’ (which I subsume into wider debates on ‘personality’), individual responsibility for loneliness, and shame. Recent literatures, I contend, leave these tensions largely unresolved, in part through a laudable attempt to avoid reproducing stereotypes and narratives which heighten the burden of shame. Thus in a recent high-profile article written by an interdisciplinary team in psychology, education, communication, and public health, we have extensive quantitative analysis of global responses on loneliness and stigma, dissected along lines of gender, age, and culture, but almost no articulation – beyond a brief note in the literature review – of what, precisely, the ‘culturally shared beliefs’ are that colour representations and experiences of loneliness, and heighten or perpetuate complex systems of stigma and shame. Although past research had demonstrated that people who feel lonely are ‘perceived to be socially inept, poorly adjusted, unlikeable and generally incompetent’, the authors note, such assumptions have been rigorously debunked (Barreto et al 2022, 2659). Far less clear (both here and elsewhere) is where these languages come from; how shame has accrued (or been assembled) around loneliness as an emotion or experience; and how this has changed over time, including during more recent (and not unclouded) attempts at destigmatisation. What is needed to understand this relationship – and far beyond the very limited scope of the present article – is a history of the lonely person as not good enough, difficult, compromised, unlovable, and morally flawed.

Barreto and her co-authors rightly argue that ‘we need to examine other ways [beyond loose cultural perceptions] in which stigma [around loneliness] can be endorsed and expressed’ (Barreto et al 2022, 2661). Indeed, scholarship in the medical humanities and social sciences has demonstrated a number of contexts in which shame and stigma have been knowingly or unwittingly produced by discourses and interventions on health (Brewis and Wutich 2019; Weil 2022; Cooper, Dolezal and Rose 2023). This body of research, I suggest, can offer a useful conceptual framework for understanding one particular way that loneliness has been invested with shame; in the deliberate acts of naming, definition, and description increasingly undertaken by a plethora of voices – including academic and clinical experts within or adjacent to the discipline of psychology – over the course of the Twentieth Century (Perlman and Peplau 1998). This is not to deny the importance and reach of lay cultural perceptions. A hint of the kinds of cultural reproduction that accompany common ways of speaking about emotions can be seen, for example, in a relatively recent discussion of Russian idioms on *odinochestvo* [loneliness or aloneness] (Pozdeeva 2011). The shared (and fluid) life and meaning that words have shapes not just how we consciously articulate our feelings and experiences, but how we actively live and inhabit them. However, that experiences of illness and low mood are framed by how they are publicly and professionally represented and discussed is, by now, an old assertion. Charles Rosenberg’s famous introduction to his 1992 edited collection with Janet Golden, *Framing Disease*, stressed the ‘role played by laypersons as well as physicians in shaping the total experience of illness’, as ‘often contentious social negotiations evoked questions of value and responsibility as well as epistemological status’ (Rosenberg 1992, xviii). Well-known historical work by Mathew Thomson has also shown how, across the twentieth century, psychological concepts and language have become increasingly central both to our self-fashioning and how we understand the world around us, including our relationships and interactions with others (Thomson 2006). Ideas which originated in relatively esoteric spaces, such as introversion and extraversion, carry particular inflections, heft, and regard which follow them through their popular life, colouring how loneliness is articulated and experienced (Bound Alberti 2018, 250). There is, in brief, no basis for disentangling experiences of shame over loneliness from past and present discourses on who becomes lonely and why.

The recurrence of personality flaw as causative story in the deliberations of the WGPW should not be surprising; it can be read, for example, in the context of inter-war work such as the psychoanalyst Karen Horney’s *The Neurotic Personality of Our Time* (Horney 1937). What is important to recognise, here, is the extent to which the Anglophone study of loneliness emerged through and alongside questions and concerns over personality, the neuroses, and social health. Often considered a foundational text, Gregory Zilboorg’s 1938 essay on loneliness for *The Atlantic* played extensively with themes of narcissism and hostility (Zilboorg 1938). Over the following decades, a proliferation of writing on loneliness in print journalism and the psy and social sciences continued to problematize personality as a decisive factor, dwelling on ‘unlovable’ character traits such as selfishness and self-pity, or difficult and distancing emotions such as anger and resentment. The act of uncovering loneliness and tracing its contours was – and is – simultaneously an act of construction^1^; this work established powerful cultural and intellectual scripts, scripts which continue to haunt recent attempts to reframe and destigmatise loneliness as an experience which ‘doesn’t discriminate’ (Department for Digital, Culture, Media and Sport 2018). Drawing on the author’s practice as a historian of medicine, this article follows the problem of ‘personality’ through primary sources in print journalism, loneliness activism, public health work, and the psy and social sciences. It traces shaming narratives on loneliness through interwar and post-war conversations on selfishness, self-pity, and the typology of lonely personalities, and considers how discourses on hostility and intolerance shaped a growing theorisation of chronic and intractable loneliness. Future work on loneliness, this article suggests, will usefully benefit from greater attentiveness and attunement to the complex psychosocial mechanisms through which shame is experienced and produced; how these (and a formidable array of other) considerations are framed and conditioned by histories of loneliness, as both an emotion and an idea; and how acute or protracted experiences of loneliness might set in motion significant changes in how sufferers engage with interventions and services, necessitating greater sensitivity to difficult emotions in restorative work (Dolezal and Gibson 2022).

## The history of loneliness

The history of loneliness is a growing field of study – situated broadly across social and cultural history, histories of medicine, the humanities, and the psy and social sciences, and the history of the emotions – with vast potential to inform, nuance, and unsettle discussions on loneliness in the present day. Although loneliness had not been a complete omission in previous historical scholarship, there has been a clear increase in research on the subject in the last decade; work which explicitly disputes the assertion that the early twenty-first century is in the midst of a loneliness ‘epidemic’ or ‘crisis’ (Bound Alberti 2018). Indeed, such intimations of present-bound crisis are themselves not new (Cooper 2023). The first steps towards the articulation of loneliness as an (at least somewhat) coherent object of historical analysis were taken by the social and economic historian Keith Snell, who outlined a potential agenda for future work alongside a spate of articles on the history of living alone (Snell 2015; Snell 2017). In the first full-length monograph on the topic, Fay Bound Alberti followed the cultural, emotional, and material history of loneliness through a series of apposite vignettes, offering valuable depth to ongoing debates on subjects such as ageing, grief, and technology (Bound Alberti 2019). Parallel research in the history of solitude, in particular the work of Barbara Taylor and David Vincent, has further interrogated the slip and play between illness and wellness in experiences of being alone (Taylor 2020; Vincent 2020). Most recently, Hannah Yip and Thomas Clifton have brought histories of scholarly loneliness into close proximity with debates on researcher mental health and the position of the humanities in present-day Britain (Yip and Clifton 2021). At the time of writing in late 2022, a Routledge handbook on the history of loneliness is in the final stages of production, for publication in early 2023; a volume of essays on early modern loneliness is being edited for Palgrave by Yip and Clifton (Barclay, Chalus and Simonton 2023; Clifton and Yip 2024). There are also (to the author’s knowledge) a number of highly promising PhD projects yet to be significantly published from, including Jeffrey Mathias’ doctoral research on experimental isolation and the cold war sciences of mind (Mathias 2022).

My own practice complements – and at times comments on – this burgeoning literature. Rather than uncovering past experiences of loneliness or historicising the processes and contexts which have made them more likely, my interest is in comprehending and critiquing how, when, where, why and by whom the *idea* of loneliness has been assembled around particular kinds of emotion and experience. Historians of loneliness, I suggest, can and should apply the kind of rigorous, critical, politically attentive questioning commonplace in historical analyses of discourses on anxiety, depression, schizophrenia, or stress, and present in recent feminist work on loneliness (Hayward 2014; Hirshbein 2009; Jackson 2013; Jones 2022; Magnet and Orr 2022). In a flawed but useful overview, the psychologists Daniel Perlman and Letitia Peplau trace the growth of Anglophone loneliness research in the twentieth century: a ‘small trickle’ of work in the inter-war and post-war periods, with only ‘a dozen or so psychologically oriented, English language publications on loneliness prior to 1960’; 64 new publications in the 1960s; roughly 170 in the 1970s; and almost 650 more between 1980 and the time of writing in 1996 (Perlman and Peplau 1998). The authors omit to acknowledge that knowledge-building on loneliness in the twentieth century took place in multiple other sites – in the arts, journalism, the humanities, charity work, and organisations such as the WGPW – but also in literatures in the psy and social sciences which addressed themes of childhood, old age, mental health, community, privacy, migration, gender roles, romance, suicide, and psychological responses to the built environment (Cooper 2018). That loneliness research proliferated significantly over the course of the twentieth century, however, is certainly true. In part, this may reflect an increased willingness on the part of researchers to classify particular experiences as loneliness, a process inextricably bound with disciplinary and professional claims to explanatory power. Modern-day loneliness research rests on this silt; while only a few ‘canonical’ early studies continue to be cited (or returned to) more than very rarely, a widespread concern with relying on outdated work means that much past research, activism, and intervention is lost to view, but remains of considerable importance in underpinning the ideas we work with today. Humanities methodologies which excavate and contextualise historical discourses on loneliness – in the case of this article, around the problem of personality – can offer significant insight into questions – i.e., why is loneliness considered shameful – which might otherwise be opaque.

## Shaming loneliness: narcissism and self-pity

Twentieth century writing on loneliness frequently devalued and shamed people who feel lonely. This process of shaming has been variously subtle, overt, intentional and accidental; it ran the gamut from knowingly attributing personal weakness and failure through to a preoccupation with individual determinants (such as specific kinds of personality) which position the lonely person as the problem, over and above the contexts, environments, and institutions which frame healthy relationships, community embeddedness, and feelings of belonging (Wilkinson 2022). One specific aim of this article is to track between historical sources where shame is a clear consequence of narrative – in other words, which are instantly recognisable as humiliating or scornful – and writing or speech which carries comparable implications, but arrives at them obliquely or with veiled meaning. To illustrate the former, we begin with a rare example of what might be termed loneliness denial, a 1929 essay in the *Derby Daily Telegraph* penned by the journalist and author of adventure novels, Andrew Soutar. Taking sight at the ‘dirge about loneliness’ that he perceived in contemporary culture and society, the essay dismissed the image of ‘lonely men and women, sobbing out their hearts in lonely corners, isolated, pariahs, unwanteds, alien to the spirit of friendship, incapable of making friends’: ‘Loneliness be hanged! Show me the man or woman who is dying of loneliness and I will show you a sufferer from the greatest evil that can afflict a race – the evil of self-pity.’ For Soutar, loneliness and ‘mental virility’ were mutually exclusive; attempted suicide turned on the ‘foolish hope’ that the pity of others would ‘lift them out of the morass into which their own lack of courage has placed them.’ Self-pity, he argued,

‘degrades those who fall back on it; nothing is so nauseating to those who are compelled to behold it… [the lonely person] makes of every friend and acquaintance a leaning-post; his lachrymose wailings sap even the physical strength of those who are courageous enough to tackle their own troubles in solitude. He asks not necessarily for money, but for pity. He is mentally lazy; he is unfitted for everything… he is a drone in the hive; he irritates the working bees’

(Soutar 1929).

Soutar’s words framed loneliness as an explicit matter of self-indulgence and moral failing, contrasting sufferers with the courageous and productive victims of their lack of emotional discipline. The self-pity inherent in loneliness, here, was ‘nauseating’ to those forced to bear witness to it; if not precisely contagious, then simultaneously a source of bodily disgust and an epicentre for social and relational malaise. Soutar’s heaping of ‘blame on shame’ prefigured shaming practices identified by Graham Scambler under post-1970s financial capitalism, in its clear preoccupation with ‘rooting out the misfits in all their heterogeneity and the variety and severity of the threats they represent’ (Scambler 2018; Scambler 2020, 2). Although it is impossible to determine the extent to which Soutar’s readership shared his feelings, other (more sympathetic) pieces on loneliness prompted deluges of letters from people who identified as lonely (Sitwell 1931; Anon 1958; Cooper 1990). It is highly plausible, therefore, that some of his audience were feeling lonely when they read these words, and that they caused or exacerbated feelings of shame.

While Soutar’s essay was remarkable in its willingness to shame its subjects, the scripts it relied on found purchase in other contemporary writing, perpetuating tropes which helped to frame loneliness as a shameful marker of spoiled identity. An article on ‘the poverty of loneliness’ written for *The Daily Mail* by the poet Edith Sitwell in 1931, for example, contrasted the loneliness ‘brought upon us by no fault of our own’ with the consequences of being ‘too selfish to gather any human beings near the fires of our heart’ (Sitwell 1931). As discussed later, this dichotomy recurred again and again in loneliness taxonomies, imposing a brittle logic of blamelessness and culpability which allowed for a particular (de)valuation of competing aetiological narratives. Sitwell’s entanglement of loneliness with selfishness drew from a well which remained undiminished for at least another three decades. In a series of letters to the editor of the *Sunday Times* in 1962, readers responded to a clutch of articles on loneliness written by the journalist (and later well-known children’s novelist) Susan Cooper; one correspondent noted that ‘it seems there is one thing wrong with the people who experience loneliness in our big cities – this prevalent habit of expecting to *get*, but omitting to *give* (Birke 1962). A second reader, identifying herself as the organiser of a social club for former psychiatric patients, wrote that ‘one point which was not made and which I regard as significant is that a great deal of loneliness is rooted in selfishness.’ Lonely people, she argued, avoided other lonely people ‘because they are terrified that the person approached may, in his or her gratitude, become a nuisance and a burden and make emotional demands.’ This second perspective represented a reformulation of Soutar’s ugly caricature of lonely people dragging those around them into a sinkhole of misery and obligation. Where the ‘mentally virile’ had been justified in maintaining their distance, however, this kind of avoidance on the part of the lonely meant they had ‘no right to grouse’ over their own suffering (Forsyth 1962). Personal responsibility for loneliness, it seems, had become a discursive script that some readers expected journalists to pay lip service to.

The uneasy proximity between loneliness and selfishness in these accounts also had traction in more formal systems of knowledge. The Ukrainian-American psychoanalyst Gregory Zilboorg is widely credited with authoring the first substantial psychological article on loneliness in the English language, in the form of an influential and much-cited piece for *The Atlantic* magazine in 1938 (Perlman and Peplau 1998). In a recurring strand of analysis, Zilboorg’s essay detailed the ‘type of sheer selfishness called narcissism’, in which the individual ‘happens to choose only himself instead of others as the object of love’ (Zilboorg 1938, 46). Loneliness, he argued, was the frequent fate of the true narcissist, those whose overwhelming self-involvement precluded their being loved by others: they ‘would feel themselves lost, they would experience a sense of uselessness, they would become low-spirited, somewhat irritable, and perhaps feel ill-treated by fate, lonely… they would find that nothing satisfied them; a feeling of emptiness would pervade them and they would appear to cower and to succumb under the burden that is loneliness.’ While loneliness often followed narcissism, the direction of travel could also be muddy and indistinct; even in ‘normal’ loneliness (or ‘lonesomeness’), he wrote, ‘we may on closer inspection discern at the beginning the germ of narcissism’, as ‘we are preoccupied with the contemplation of our own misery.’ Self-pity, here, was a facet of self-love, rather than a contrary or dissonant experience (Zilboorg 1938, 47). As a brief example of how knowledge is built, we can see Zilboorg’s hypotheses cited directly in another foundational text, the psychiatrist Frieda Fromm-Reichmann’s famous 1959 essay (also titled ‘Loneliness’). While Fromm-Reichmann gave far less space to narcissism than Zilboorg, she rephrased one of his key conclusions relatively uncritically, noting that the ‘narcissistic- megalomaniac attitude will not be acceptable to the environment, which will respond with hostility and isolation of the narcissistic person.’ While this reframing shifted emphasis away from the individual and towards a rejecting environment, the process was nonetheless framed as natural and inevitable (Fromm-Reichmann 1990, 312). Interestingly, Fromm-Reichmann speculated further that ‘the lonely person may be displeasing if not frightening to his hearers, who may erect a psychological wall of ostracism and isolation about him as a means of protecting themselves’; again, the emphasis is shifted to the reactions of others, but this can still be read as another iteration of Andrew Soutar’s depiction of the lonely as sources of emotional contamination (Fromm-Reichmann 1990, 314).

Loneliness, in these accounts, became a cipher to be decoded; it marked the sufferer out, offering (frequently false) insights that they hadn’t intended to share. One comment made by a young person to researchers from the Co-op foundation in the late 2010s is pertinent here: ‘to be seen as lonely is to be seen as though there’s something wrong with you’ (Co-op 2019, 8). The recurring use of ‘to be seen’ in this testimony draws focus specifically to the relationship between loneliness and shame. The emphasis here is on the regard of others; identifying as lonely is exposing and fraught with danger, not just because loneliness is a culturally devalued and shamed experience, but because it is impossible for the person disclosing to exert full control over how that admission is interpreted and made salient. When narratives on loneliness locate aetiological power in personal traits and failings, these forms of knowledge work backwards on the subject, colouring lived experiences with both active and anticipated shame. This is not to say that mid-century taxonomies of loneliness were unable to take structural factors seriously into account; indeed, frequently the opposite (Cooper 2023). The persistence of narratives of individual responsibility around and alongside articulations of collective problems such as housing, community, poverty, gender roles, and work, however, helped undermine loneliness as a language of political and environmental complaint; a lens which could have allowed for a decreased burden of shame. This ambivalence can be seen in attempts to provide exhaustive lists of the reasons why and how people became lonely. In 1959, for example, the National Union of Townswomen’s Guilds (NUTG), a former suffragist organisation with an increasing emphasis on citizenship and education, sent out guidance on loneliness to their regional branches; alongside accounts of migration, urban anonymity, and old age, one sub-heading was given over to ‘misfits’, one of ‘the pathetic problems of most communities.’ Noting that ‘in many cases people… are not liked because of faults of character’, the section described the ‘ill-adjusted, the troublesome, the bores, the gauche and the not-so-likeable, most of whom would dearly like to be liked’ (National Union of Townswomen’s Guilds 1959, 4). This alights on an enduring point of tension in loneliness narratives. Even when external causative stories are acknowledged, individual characteristics are difficult for us to extricate, particularly bearing in mind that feeling ‘not-so-likeable’ is a common dimension to many (if not most) experiences of loneliness. Even when our experiences of loneliness are wholly explained by contexts far beyond our control, whether on the level of individual circumstances and structural inequalities, or the deeper philosophical plane of alienation as the default mode of modern (or even just human) life, what we might term *the loneliness of loneliness* muddies and obscures our ability to reckon with it as a collective plight. Any narrative which takes likeability or lovability seriously as an objective or measurable trait contributes to this muddiness, inviting a shaming interpretation of individual failure when far less individualising narratives more than suffice.

## Chronic loneliness: hostility, intolerance, and ‘the snub’

As the philosophers of emotion Tom Roberts and Joel Kruger have recently argued, representations of loneliness as revolving around a felt absence of longed-for social goods are complicated by experiences of chronic loneliness; what happens, they ask, when the desire for connection seems to fade? (Roberts and Krueger 2020). This tension can be read in the empty space around ‘most of whom’ in ‘most of whom would dearly like to be liked’, although the semblance of disinterest in friendship or help can also be a means of saving face (National Union of Townswomen’s Guilds 1959, 4). Post-war writers on loneliness were clear that loneliness had a specific temporal prognosis, becoming increasingly severe and resistant to disruption. Friendly overtures, recruitment to community groups, and exposure to services, consequently, had to be timed before damaging and distorting processes of ‘social encystment’ could take full effect, narrowing the window of intervention to a scant matter of years (Pearse and Crocker 1943, 248; Cooper 2023). Contemporaries identified an enervating social atrophy at work in longer experiences of loneliness; as Susan Cooper put it, a ‘tortured inaction, the penalty for a life which has tightened into a hopeless circle’ (Cooper 1962b; Eckersley 1958). Frequently, journalistic representations of loneliness turned on the conceit of uncovering hidden suffering, with ‘great outpourings of pent-up loneliness’ occurring whenever interest or sympathy was shown (Jeffery 1961). The language of recognition and restitution, however, imposed a new narrative arc, offering the opportunity to dissipate shame by demonstrating willingness to return, to re-adopt the role of the healthy, connected, social citizen.

What, then, about the dissenters, the suspicious, antagonistic, ungrateful, or reluctant to change? For Gregory Zilboorg, just as loneliness was ‘intimately related to man’s narcissism’, so ‘narcissism [carried] with it more than a seed of malice, of hostility… the nucleus of hostility, hatred, impotent aggression of the lonely and abandoned’ (Zilboorg 1938, 49/53). Likewise, in their 1959 communication to their branches, NUTG stressed that ‘some of the lonely ones, through sheer loneliness, have become bitter, queer, or arrogant’ (National Union of Townswomen’s Guilds 1959, 2). Particularly in discourses on long and intractable experiences of isolation, lonely people’s hostility – and their capacity to tolerate seeming flaws in others – became an increasingly vexed question. One committee member of the WGPW, for example, identified a refractory core of ‘problem people’ for whom it is ‘sheer misery to meet strangers… who will only have social life on their own terms, who even shrink from or snub neighbourly advances… [who] will not pay the price in effort and tolerance’ (Morrison 1955). These narratives demarcated moral space between the deserving lonely, the victims of external pressures and circumstances, and those whose loneliness could be construed as a problem of their own recalcitrant nature.

The author Evelyn Home’s address to the WGPW, here, bears returning to at greater length. Her usual mode of communication – agony aunt columns and the growing genre of self-help – imposed a specific perspective on loneliness, emphasising individual responsibility for health in ways which have been rigorously critiqued as creating fertile ground for shame (Dolezal and Spratt 2022). For Home, the path away from loneliness began with ‘a bit of self-knowledge.’ Through her magazine work, she was frequently in receipt of letters from readers, many of which outlined their own feelings of loneliness or relational dilemmas. In her address to the WGPW, these testimonies became texts for Home to interpret; as above, in their admission of loneliness, narrative control had been lost. Reading between the lines of her readers’ letters, here, was a means for Home to create (and impart) unwilling knowledge about the lonely, in what can be read as a shaming and extractive way:

If I get a letter from a woman who says that her family shun her, I usually find in the letter the germ of why they shun her, and often it is because she is intolerably selective; she likes only a certain type of person… the person she thinks she likes does not exist; such a tiny person whose whole interest is concentrated on the lonely person; there is no person as small as that.

For Home, the inherent self-involvement of the lonely precluded their seeing others as anything more than two-dimensional sources of support or relief; they constructed an impossible archetype of the selflessly giving lover, sibling, parent, child, or friend, and refused to countenance anything less. The non-lonely, she argued, are ‘snubbed over and over again by lonely people’:

A lot of them decide to remain lonely: decide that the things we tell them to do are nonsense – they are not that sort of person. The lonely person writes this sort of letter: “I am desperately lonely: I have not a friend existing. Do not tell me to get in touch with my family, to join a club, do social work, etc., because I abhor hospitals, or dirt, or doing good.”

Home’s imagining of how people became and remained lonely conjured a depoliticised social world where individual decisions to opt in or out were the primary determinants of experience, and a stubborn unwillingness to participate in the pageantry of rescue was clear evidence of the difficult personality that had kept others away in the first place. The snub, here, was a means to achieving a kind of warped and fleeting significance, of wresting a reaction – and, with it, some form of acknowledgement – from the disliked (but coveted) other: ‘someone is, as it were, affected by their presence’ (Home 1955).

Parallel discourses on loneliness, intolerance, and hostility, particularly among older adults, can also be read in the annual reports that Medical Officers for Health (MOH) delivered on public health activities in London boroughs; while at times more sophisticated, they nevertheless continued to problematize lonely subjects in uncomfortable ways. In the 1950s and 1960s, MOHs – public health officials with jurisdiction over relatively sizeable areas – drew complicated – and sometimes cyclical - relationships between loneliness, neglect, personality deterioration and dementia, cataloguing the difficulties that loneliness in old age presented to health visitors and domestic help services.^2^ Reports covering two boroughs, Southwark and Leyton, are instructive here. In the first instance, the MOH for Southwark, A.D.C.S. Cameron, wrote in 1959 that loneliness could precipitate ‘a physical and mental decay and disintegration which leads to the rapid onset of a really senile state, when all self respect is lost. Old people then become careless about themselves; they neglect their personal appearance and make no attempt to take proper meals, and mentally then soon become hostile and suspicious’ (Cameron 1959, 60). A year later, he noted ‘how often… an almoner or a doctor ring[s] us asking for services to an old person, adding "She lives alone" or "She feels so lonely." And yet sometimes the lonely are the most difficult to approach’ (Cameron 1960, 58). In Leyton in 1958, Melville Watkins repeated the distinction – which, as above, imposed an unsubtle hierarchy of deservingness and need – between ‘those who are cut off from their contemporaries through circumstances over which they have little or no control and those who are lonely by temperament.’ In the latter case, he argued, ‘the greatest difficulty arises, and the most time consuming cases are those elderly people who through their eccentricities either refuse help or obstruct when it is granted’ (Watkins 1958, 9). The dehumanising language of obstruction, and of people ‘consuming’ time, posed its subjects as burdensome, a strain on vital services, in ways which ran in parallel to shaming post-war discourses on obesity, hypochondria, or time-wasting in the NHS (Bivins 2020; Moore 2022). These themes came even more forcefully to the surface in a report written by Watkins’ successor as MOH for Leyton, E.W. Wright, on a training course for domestic help in 1963:

The first session began with group discussion on the question "What do you find most trying in dealing with old people?" which was followed by plenary discussion of this subject with the Area Medical Officer. Some of the subjects emerging included the way old people follow the Help about, how they do not understand present-day conditions or prices, forgetfulness, suspicion or general nastiness perhaps related to jealousy of the Home Help's health and strength, and a very wide variety of interesting and at times amusing difficulties… after a cup of tea two members of a local dramatic society acted a comedy sketch of a dialogue between an old lady and a Home Help, in which discussion points were highlighted as follows:-

(a) The old lady wants things done in a particular way.
(b) She is suspicious of theft.
(c) She keeps too much furniture to make cleaning easy.
(d) She is unwilling to accept the help of neighbours.
(e) She will not eat what she is not used to and has to be persuaded.
(f) Loneliness makes her talk so much it is difficult for the Help to get away.
(g) Some old people are scroungers and feel they ought to get more than they do (Wright 1963, 50-51).

Unfortunately, Wright did not record whether the group roleplayed any solutions to these dilemmas, or what the helpers present were supposed to take from the session (beyond the forewarning that the people in their care could be suspicious, obstinate, particular, or otherwise unpleasant and difficult to manage). In the narratives on chronic loneliness explored in this article, the question of how stubborn or hostile sufferers might be restored to some degree of social or relational health revealed a spectrum of approaches; laissez-faire abandonment, infantilising paternalism, and a liberal preoccupation with acts of individual kindness. One member of the WGPW, E.J.D. Morrison, suggested that ‘if such people with their eyes open [emphasis in source] choose loneliness rather than a social life not wholly congenial, well and good. Their lives are their own’ (Morrison 1955). On the topic of ‘misfits’, NUTG noted that, while people were usually not liked through their own fault, ‘it is no use trying to absolve ourselves of responsibility on these grounds. They are like a child who has climbed a tree and can move neither up nor down; no use telling the child that he should not have got himself into such a position!’ (National Union of Townswomen’s Guilds 1959, 4). For Evelyn Home, the answer lay in a stoic refusal to be deterred or offended, which could eventually draw the sting from the preferred weapon of the lonely, the snub: ‘it is the time to sink pride to the lowest level to which it has ever gone. Be snubbed over and over again… the snubs cease to mean anything at all, and soon they cease to render any sort of amusement’ (Home 1955). In this imagining, the answer to individual failure was individual exceptionalism, with kindness, good citizenship and charity work counterbalancing the resentment and withdrawal of the chronically lonely; as Cooper put it in 1962, there was a shared responsibility to ‘bring into the circle of humanity those shadows of men and women who have time only for themselves’ (Cooper 1962a; Anon. 1956).

## Loneliness and shame

How can these old threads help us make sense of the relationship between loneliness and shame in the present day? In 2018, when Theresa May’s Conservative government unveiled its ‘loneliness strategy’, a development tied to the headline-grabbing ‘minister for loneliness’ earlier in the same year (in reality an addition to the portfolio for the Department for Digital, Culture, Media and Sport), attention rightly turned to the hypocrisy of performing a highly visible concern with loneliness at the same time as presiding over a deeper unravelling of the welfare state and our social and community infrastructure, under the guise of austerity (Department for Digital, Culture, Media and Sport 2018; Stenning and Hall 2018). However, the more striking point was that loneliness had become an idea that a government like that could do business with; i.e., more or less completely stripped of its potential to act as a radical language of critique, at least in the broader public sphere (Wilkinson 2022). We were left with Evelyn Home’s impoverished imagining of loneliness as an experience unmoored from structural problems, political decisions, social changes, or big historical, cultural, and ideological shifts. Despite valiant attempts to the contrary, loneliness had been recast as an apolitical emotion; a development which, in absolving powerful vested interests from responsibility for the problems it causes, has of course been profoundly political.

Shame matters here for two reasons. In the first instance, this article has, through a narrow snapshot of discourses on loneliness and personality, demonstrated one way in which shame has been assembled around the experience, setting long cultural scripts in motion. These have persevered in the stories we tell about loneliness, likeability, and individual failure, but also in academic research on loneliness, selfishness, anger and aggression, particularly in the longstanding work of Ben Lazare Mijuskovic (Check, Perlman and Malamuth 1985; Stober 2003; Mijuskovic 1979; 1988; 2015; 2019). Indeed, Barreto and her collaborators cite a number of studies which began from the assumption that lonely people really *are* socially inept (Barreto et al. 2022, 2659). Across the mid-late twentieth century, neoliberal logics of individual responsibility have also fed an increasing preoccupation with lifestyle and behaviour in public health, framing an ideal of the healthy, well-connected, competitive self which explicitly excludes and shames loneliness (Bivins 2020; Cooper, Dolezal and Rose 2023).

In the second instance, attempts at de-stigmatisation have been an important contributor to narratives on loneliness which, counter-intuitively, allow shame to go unaddressed. Destigmatisation (not just for loneliness) has a number of important histories, unfortunately beyond the scope of the present work. An early article on loneliness and stigma appeared in *The Times* in 1971, setting itself pointedly in tension with some of the narratives discussed above: ‘an admission of loneliness is seen by many as an admission of failure… loneliness still carries a stigma, and the lonely are often told that they are being self-pitying and introspective.’ Interviewing Pamela Warren, the general secretary of the Camden Council of Social Service, the piece quoted her assurance that ‘we do not dismiss loneliness as selfishness’; indeed, as her colleague, Richard Barr, explained, ‘we are hoping to get the point across that loneliness is not something to be ashamed of (Hunter Symon 1971). Historically, social services have had a complex and ambiguous relationship with shame, and this is one reason why recent work on shame-sensitive practice in human services has emerged in part from this perspective (Dolezal and Gibson 2022). While Warren and Barr’s work addressing shame around loneliness in community settings seems as though it might have been genuinely constructive and useful, public discourses on loneliness and shame in the 2010s and 2020s have demurred from a rigorous confrontation of how the two interrelate, occupying themselves instead with the sanitised assertion that ‘loneliness doesn’t discriminate.’

Originating as a quotation from the Labour MP Jo Cox – ‘young or old, loneliness doesn’t discriminate’ – which was intended to shift conversations on loneliness to encompass the entire life course, this phrase has become firmly embedded in the lexicon of charities, government strategy, and politicians of all stripes (Department for Digital, Culture, Media and Sport 2018; Reeves 2017). Eliding the well-evidenced connections between loneliness and a raft of health and social inequalities, the aphorism distils a kind of bland (but pernicious) de-stigmatisation, in which problems which are often direct matters of exclusion and injustice could supposedly happen to anyone (Wilkinson 2022). While this may seem like an antidote to the explicitly shaming imaginaries which connected loneliness with ‘unlovable’ personality traits such as selfishness or hostility, it effectively wallpapers over the only causative story with the narrative power to contest them. In the process, it draws focus away from the systemic forms of shaming which are produced by entrenched inequalities, and which frequently result in loneliness and alienation (Jones 2022). As Xiaoqi Feng and Thomas Astell-Burt have – somewhat charitably – put it, these are ‘the shortsighted, inadvertant, reckless, and negligent decisions made across society, fostering stigma and structural discrimination (racism, sexism, ableism, classism), that generate social and built environments that make many people with these characteristics feel perpetually isolated and unsafe’ (Feng and Astell-Burt 2022).

What is needed – and what the historical sources discussed in this article provoke – is a more nuanced engagement with how shame and loneliness interact. De-stigmatisation and sustained attentiveness to shame are not the same. The attitudes and behaviours that inter- and post-war writers found evident of selfishness, suspicion, hostility, intolerance, and stubbornness – serious personality flaws that made chronic loneliness explicable – might better be understood as reasonable reactions to, for example, condescending and dehumanising interactions with services, but also as complex mechanisms of shame, anticipated shame, shame-avoidance, and attempts to save face. For the phenomenologist Luna Dolezal, long experiences of shame are ‘alienating, isolating and deeply disturbing… [provoking] powerful feelings of despair, inferiority, powerlessness, defectiveness, and self-contempt’; shame can also go ‘underground’, masking itself in ‘self-hatred, anger, excessive pride, [and] insecurity.’ Dolezal describes a specific experience that she terms ‘explicit chronic shame’, which could be one of the best tools we have for understanding how loneliness reproduces itself over time, becoming increasingly resistant to outside interference. With explicit chronic shame, ‘the horizons of one’s present experience come to be dominated by the possibility of shame; it feels as though shame is constantly just around the corner’ (Dolezal 2022). When every remedy for loneliness pivots on some form of social contact, at the same time as the systemic shaming of loneliness as a matter of personal failing explicitly undermines confidence in our ability to successfully navigate social situations, the anticipation of further shame can trigger powerful defensive strategies. We can see these at play, for example, in the exaggerated excuses of Evelyn Home’s archetypal ‘lonely person’ who protested ‘do not tell me to get in touch with my family, to join a club, do social work, etc., because I abhor hospitals, or dirt, or doing good’ (Home 1955). This problem is a persistent one, colouring how lonely people interact (or don’t) with services and community resources, as well as estranged or distanced friends or relations. Closer attention to the shame of loneliness (and the loneliness of shame), as well as the experiences and histories which tangle the emotions together, has vast potential to transform and energise our understandings of both.

## Footnotes

1 This is, of course, also true of shame, another idea which has been rarely adequately historicised. To do so is beyond the scope of this work, but readers interested in the history of shame might fruitfully start with Peter Stearns’ Shame: A Brief History (2017).

2 For an excellent – and fully searchable – digitised resource, see https://wellcomelibrary.org/moh/
